# Find your way: Building an atlas from transcription factor activities in the Marchantia gemma

**DOI:** 10.1093/plcell/koae061

**Published:** 2024-02-27

**Authors:** Gwendolyn K Kirschner

**Affiliations:** Assistant Features Editor, The Plant Cell, American Society of Plant Biologists; The James Hutton Institute, Invergowrie, Dundee DD2 5DA, UK

**Figure 1.**
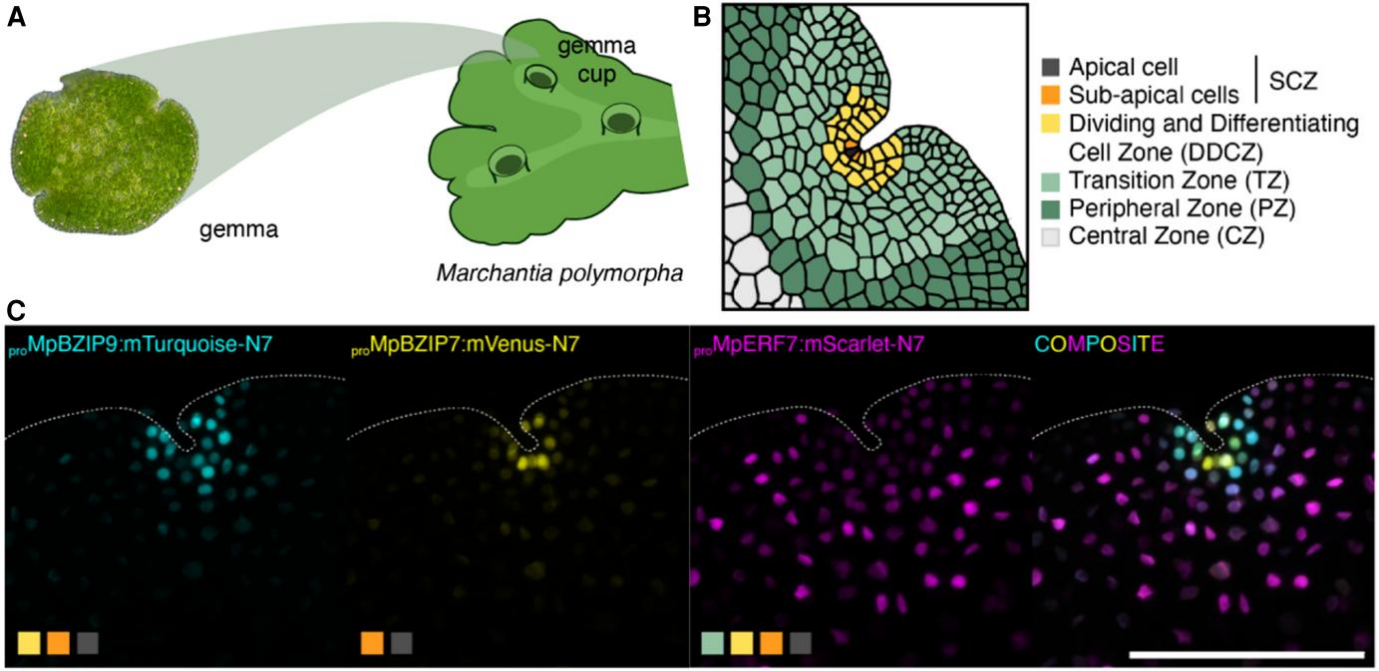
Cell identities of Marchantia gemma tissue revealed by transcription factor promoter activity. **A)** Marchantia thalli give rise to gemma cups, which contain the small, disc-like gemmae. **B)** Cell types in Marchantia gemma apical region. **C)** Expression pattern of multiple fluorescent reporters of TFs in the apical region of Marchantia gemmae (color blocks correspond to panel **B**). Adapted from Romani et al. (2024), Figures 1, 4, 5.

Liverworts are often found in moist places worldwide, especially in humid tropical regions, in damp wooded areas on tree trunks, damp soil and rocks, and in gardens. At first glance, their organs resemble root- and leaf-like structures of vascular plants. But in contrast to vascular plants, which have a dominant diploid (sporophytic) life form, liverworts are mostly haploid (gametophytic) throughout their life cycle (Kohchi et al. 2021). The haploid spores divide and form a leaf-like lobe, the thallus. On its surface, the thallus forms gemma cups that contain gemmae, small discs with lateral, slightly indented apical notches (Fig. 1, A and B) (Kohchi et al. 2021). Similar to the shoot apical meristem of vascular plants, liverworts maintain a stem cell population in the vegetative gametophyte meristem, but to date we do not know much about its regulatory networks.

The liverwort Marchantia (*Marchantia polymorpha*) is commonly used for genetic and developmental studies because the genome encodes only about 450 transcription factors (TFs), about one-fifth of the number of Arabidopsis TFs (Bowman et al. 2017). Several TF subfamilies have only a single family member and can be easily studied without facing redundancy issues. **Facundo Romani and colleagues** (Romani et al. 2024) systematically isolated a majority of the promoter elements of Marchantia TFs and mapped the expression pattern of transcriptional reporters to the gemmae. The authors extracted the 5′ untranslated regions together with adjacent upstream sequences from putative TF-encoding genes, covering about 82% of all TF genes in the Marchantia genome. They visualized the expression patterns of the transcriptional reporter lines by confocal microscopy, normalizing the expression strength to generate a linear vector that comparably represented the expression patterns of different reporters. From that, they identified 5 clusters with different expression domains, either with a peak at the apical notch position, the central zone, or evenly distributed expression.

Based on the observed expression patterns, the authors made a map of different cell types across the Marchantia gemma (Fig. 1B) so that every observed expression pattern could be classified as active in one or a combination of cell types. By clustering the expression domains and cell types, the authors reconstructed putative developmental pathways for cell differentiation during gemma development. The authors identified promoters expressing in the apical notch and other zones, such as the dividing and differentiating zone, the transition zone, the peripheral zone, and the stem cell zone. They also identified zones in the gemmae that have not previously been defined, such as the border cells, consisting of 2 to 3 layers at the margin of the gemmae, and the attachment point cells, the cells connected to the gemma cup before detachment of the gemmae.

Romani and colleagues followed the expression of reporters active in the cells in the stem cell zone in the apical notch in more detail (Fig. 1, B and C). The stem cell niche is proposed to consist of one apical cell and derived sub-apical cells, which could function as transit-amplifying cells for the proliferation of future differentiating cells (Kohchi et al. 2021). They identified promoters that were specifically expressed in either the apical cell or the sub-apical cells. Following their expression supported the hypothesis that cell differentiation occurs adjacent to the apical cell. However, it suggested that the gene regulatory network that forms the apical meristem in bryophytes like Marchantia is not homologous to the one in vascular plants. Following expression patterns over time indicated that cells of the division and differentiating zone, the transition zone, and the peripheral zone expanded and divided in the first days of gemma development to maturation. However, only cells of the dividing and differentiating zone actively expanded and divided during cell differentiation, while transition and peripheral zone expanded to form the boundaries of the apical notches.

With their study, Romani and colleagues provided a collection of promoters for all cell types in the Marchantia gemmae in a publicly available database and thereby a new tool to study vegetative development. This “traditional” approach was able to detect features of cellular organization and gene regulation in the apical meristem that had not been resolved by time-resolved single-cell RNA-seq (Wang et al. 2023). The reporters can be used as markers to visualize the dynamics of specific cell types over time and isolate cell type-specific nuclei or to drive gene expression of genes of interest to genetically manipulate the tissue.
